# Bone growth as the main determinant of mouse digit tip regeneration after amputation

**DOI:** 10.1038/s41598-019-45521-4

**Published:** 2019-07-04

**Authors:** L. A. Sensiate, H. Marques-Souza

**Affiliations:** Department of Biochemistry and Tissue Biology, Universidade Estadual de Campinas, São Paulo, Brazil

**Keywords:** Histology, Regeneration, Regeneration, Histology

## Abstract

Regeneration is classically demonstrated in mammals using mice digit tip. In this study, we compared different amputation plans and show that distally amputated digits regrow with morphology close to normal but fail to regrow the fat pad. Proximally amputated digits do not regrow the phalangeal bone, but the remaining structures (nail, skin and connective tissue), all with intrinsic regenerative capacity, re-establishing integrity indistinguishably in distally and proximally amputated digits. Thus, we suggest that the bone growth promoted by signals and progenitor cells not removed by distal amputations is responsible for the re-establishment of a drastically different final morphology after distal or proximal digit tip amputations. Despite challenging the use of mouse digit tip as a model system for limb regeneration in mammals, these findings evidence a main role of bone growth in digit tip regeneration and suggest that mechanisms that promote joint structures formation should be the main goal of regenerative medicine for limb and digit regrowth.

## Introduction

Regeneration is the process in which lost tissues are aesthetically and functionally re-established^1,2^. In a classic regeneration model observed in the limbs of salamanders, the regenerative process includes bone, muscle, tendons, joints, nerves, blood vessels, mesenchyme and epidermis. After amputation, lineage restricted stem cells migrate to the central distal-most region of the limb and compose a structure called blastema. The salamander blastema can reconstitute the limb with identical form and function to an unamputated limb, as many time as needed^3^. Regeneration on mammals has been reported for the seasonal growth of deer antlers^4^, ear hole closure in rabbits and mice^5,6^ and during digit tip regeneration in humans^7,8^, monkeys^9^ and mice^10–12^.

Mouse digit tip is one of the best-characterized models for tissue regeneration in mammals. Amputations that eliminate up to 50% of the terminal phalanx are considered distal amputations and result in the recovery of the general morphology after about 30 days, resembling non-amputated digits^13–15^. However, studies show that the reconstituted mouse digit tips never re-establish the original size^13^. Unlike limb amputation in salamanders, distal amputation in mice affects only the skin and the distal portion of bone and nail, without damaging muscles, tendons, glands or joints (Fig. Supl.1, line b). When amputations occur more proximally, eliminating over 60% of the mouse digit tip (Fig. Supl.1, line a), the wound heals as expected, but the digit tip does not regrow^15^.

The main structure shaping the mouse digit tip in size and form is the terminal phalangeal bone (Fig. Supl.1). As all long bones, the terminal phalanx is formed during embryonic development through endochondral ossification, resulting in longitudinal growth, and through the process of appositional ossification, resulting in peripheral growth^13^. Differently from all long bones, the length of the distal phalanx is further increased by an additional ossification center located at the distal tip of the bone, through intramembranous ossification^13,14^. Estimations point that 55% of the postnatal elongation of the distal phalanx of mice is a consequence of this distal process^13^. One study showed that although distal amputation eliminates part of the terminal phalanx formed by endochondral ossification, bone regrowth after amputation is exclusively due to distal intramembranous ossification^13^. Similar to bone formation by intramembranous ossification during development or after injury^16^, bone regrowth after distal amputation of the mouse digit tip depends on Wnt signaling^17^.

Re-establishment of homeostasis and nail and bone regrowth are expected to occur naturally after digit tip lesions. However, the formation of a blastema, which is a hallmark of epimorphic regeneration in salamanders, has been largely discussed in mammals^7,9,18–22^. The search for a mammalian blastema is sustained by the idea that, as in salamanders, tissue-specific cells, or stem/progenitor cells, would respond to the distal amputation lesion, migrating to a central distal-most region of the digit, creating the multi-tissue structure comprising the regenerating digit. A study in mice^17^ shows that as in salamanders, each tissue comprising the regenerated digit is formed by tissue-specific stem cells residing in the tissues preserved after amputation, suggesting that trans-differentiation does not occur in amputated mouse digit tip. However, it is not known whether these stem cells are integrating a regenerative blastema induced by the distal amputation or simply generating more tissue through endogenous tissue repair responses.

In this study, we compared digit regenerative capacity after distal and proximal amputation and propose a hypothetical mechanism by which digits are able to regain a morphology that is close to normal after distal amputations but fails when amputations are performed proximally. While most tissues re-establish homeostasis in very similar ways in distal and proximal amputated digits, bone growth is only observed after distal amputations. Observing the regions affected by each amputation plan, we propose that the main difference between these two amputation plans is the elimination of osteogenic signals and precursor cells in proximally amputated digits. In distally amputated digits, the source of osteogenic signal emanating from the nail^17^ and the presence of osteoprogenitor cells in the periosteum^23^ could be sufficient to promote bone growth and give the digit a new tip.

## Results and Discussion

### Mice do not regenerate digit fat pad

**Figure 1:**
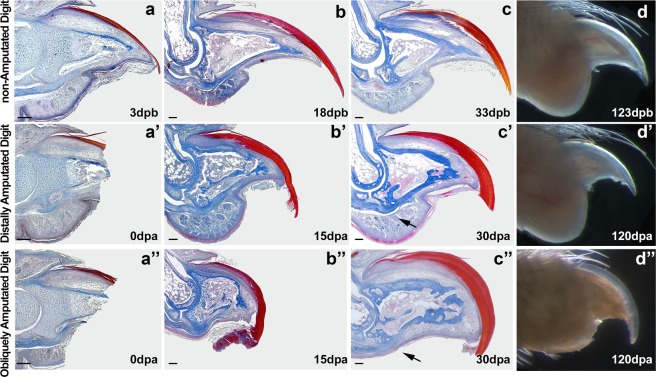
Normal and amputated mouse digit tips showing the completeness of tissue regeneration after amputation. We performed the amputations at 3 days after birth (dab). Non-amputated digit sections at 3 (**a**), 17 (**b**), 33 (**c**) and 123 (**d**) dab were used as control for comparison. Digit tips after distal amputation at 0 (a’), 15 (b’), 30 (c’) and 120 (d’) dpa. Digit tip after oblique distal amputation at 0 (a”), 15 (b”), 30 (c”) and 120 (d”) dpa. Mallory trichrome stain (a – c”) and bright field images (d – d”). Arrows in c’ and c” indicate the presence and absence of the fat pad, respectively (Scale bars, 100 μm).

Our analysis began questioning whether all tissues and structures composing the digit tip would regenerate in distal amputations, as previously described. The fat pad is a multi-tissue structure dorsally located in the digit tip and usually it is not affected by distal amputations, which are usually performed at 90° of the proximal-distal axis of the digit (Fig. Supl.1, line b; Fig. 1a’). We performed an oblique distal amputation by tilting the amputation plan to 120° of the proximal-distal axis of the digit (Fig. Supl.1, line c; Fig. 1a”). Both types of amputation remove the distal end of the phalangeal bone and the nail, however, the fat pad is removed only in digits that suffered oblique amputation. At 30 days post amputation (dpa), digits that suffered distal amputation (Fig. 1c’) or oblique amputation (Fig. 1c”) reconstituted the nail organ, the distal portion of the terminal phalanx and the gross digit morphology, as previously reported. However, digits that suffered oblique amputation do not reconstitute the fat pad (compare arrows in c’, c”, Fig. 1). This difference can also be seen at 120 dpa, when both amputated digits have nearly identical external morphology as non-amputated digits, except for the absence of the fat pad in obliquely amputated digits (compare Fig. 1d,d’,d”).

These results corroborate the findings of one study^19^ that despite suggesting a regenerative capacity of the fat pad, reports that the eccrine glands are uncapable of regenerating. This observation leads to two possible regenerative scenarios. First, it is possible that mouse digit tip is capable of epimorphic regeneration^12,24–27^. In this scenario, amputations would lead to the formation of a blastema structure that results in the formation of all missing parts of the digit, such as the nail, the mesenchyme, the terminal phalanx and the fat pad. Supporting this, one study^17^ demonstrated that bone regrowth after distal amputations occur through the formation of a blastema, while some authors^19,28^ reported a regenerative capacity of the fat pad in mice. However, the blastema tissue described by one study^17^ was characterized only by its capacity to differentiate into bone tissue. Furthermore, some findings on the regenerative capacity of the fat pad^19,28^ report the absence of eccrine glands.

The second scenario is based on the intrinsic capacity to regenerate upon lesion of each tissue of the digit tip affected by distal amputations; epidermis^19,29^, bone^30,31^ and nail organ^32^. This scenario is further corroborated by our observations that the main processes involved in the regeneration of the amputated digit tip, namely clot formation, re-epithelialization and re-amputation, seem to be indistinguishable between proximally amputated digit and distally amputated digit.

Attempting to distinguish between these two possibilities we compared distally amputated digits and proximally amputated digits to non-amputated tips for the main processes of regeneration: clot formation, re-epithelialization, re-amputation, blastema, nail and bone formation.

**Figure 2:**
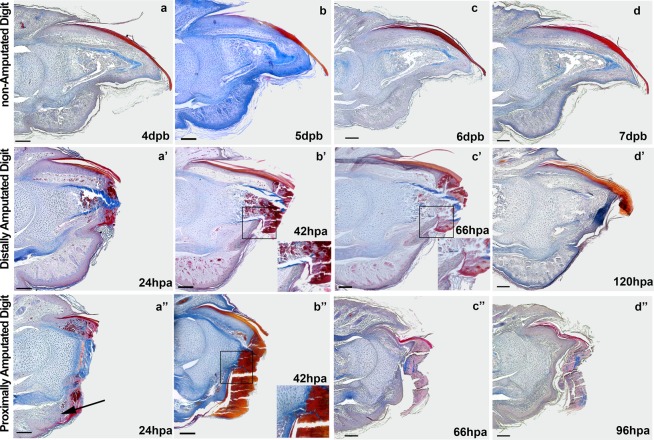
Histological characterization of re-epithelialization and clot formation in sagittal sections of distally and proximally amputated digits. Non-amputated digits at 4 (**a**), 5 (**b**), 6 (**c**) and 7 (**d**) dab were used as control. Distally amputated digits after 24 (a’), 42 (b’), 66 (c’) and 120 (d’) hpa. Proximally amputated digits after 24 (a”), 42 (b”), 66 (c”) and 96 (d”) hpa. Clot covering the entire extension of the lesion after 24 hpa, isolating the healthy tissue of the digit from the external environment on distally amputated digit and proximally amputated digit (a’ – a”). Elimination of the tissue invaded by clot after completion of re-epithelialization, taking place 96 h after proximal amputation (d”) and 120 h after distal amputations (d’). Insets in b’ – b” show that re-epithelialization halts in the ossified portion of the terminal phalanx in the distally amputated digit and in the cartilaginous portion of the phalanx in the proximally amputated digit. Mallory trichrome stain. (Scale bars, 100 μm). Amputations performed at 3 days after birth (dab).

Clot formation and re-epithelialization are commonly observed after lesions in mammals. The histological characterization of clot formation in distally amputated digit and proximally amputated digit in the first days after amputation showed similar temporal and morphological patterns in both amputation plans (Fig. 2). At 24 hours post-amputation (hpa) we can observe clot tissue covering the entire extension of the lesion on the distally amputated digit and proximally amputated digit (Fig. 2a’ – a”).

A delay in the process of re-epithelialization in distally amputated digits has been correlated to a regenerative response^25^. We followed tissue dynamics over time in distally amputated and proximally amputated digits and noticed that the path of re-epithelialization in distally amputated and proximally amputated digits is composed by different tissues. Re-epithelialization in distally amputated digits occurs through an ossified portion of the terminal phalanx (Fig. 2b’), in proximally distal digits re-epithelialization occurs through a cartilaginous tissue (Fig. 2b”). Interestingly, in both amputations re-epithelialization starts at the same time at the extremities of the lesion, migrating towards the center of the wound (Fig. 2a’ – a”) and reaching. the ventral side of the phalanx before 42 hpa (Fig. 2b’ – b”). However, while keratinocytes reach ossified tissue in distally amputated digits (Fig. 2b’, detail), keratinocytes reach cartilaginous tissue in proximally amputated digits (Fig. 2b”, detail). The process of re-epithelialization halts in both forms of amputation in this moment. At around 66 hpa, migrating keratinocytes in distally amputated digits are still halt at the ventral side of the phalanx (Figure. 2c’), while keratinocytes in proximally amputated digits have already migrated through the terminal phalanx almost entirely (Fig. 2c”). As a consequence, the epithelium closes at 96 hpa in proximally amputated digits, while closing in distally amputated digits occurs approximately 24 hours later (Fig. 2d’ – d”). Therefore, there is the possibility that the ossified tissue would form a more resistant physical barrier to migrating keratinocytes than the cartilage tissue in proximally amputated digits. This could mean that the approximate 24-hour delay of distally amputated digits to complete re-epithelialization is a consequence of the longer time necessary for keratinocyte invasion in the ossified portion of the phalanx.

Another interesting observation regarding re-epithelialization is the establishment of a new and more proximal amputation plane in both, distally and proximally amputated digits. We named this process ‘physiological amputation’, to distinguish it from the physical amputation performed with the scissors. Another group^33^ also observed this process in the amputated digit tip of adult mice and suggested that this is an evolved response linked to mechanisms controlling the regeneration in mouse digit tip. However, as observed in Fig. 2(a’ – d”), the process of physiological amputation is very similar to distally and proximally amputated digits. As described above, in both amputated digits, epithelial cells migrate in a path proximal to the clot (Fig. 2b’ – b”), halting when they reach the terminal phalanx (Fig. 2b’ – b”). While proximally amputated digits finish re-epithelialization with 96 h, distally amputated digits finish the process only 120 h after amputation, probably due to the longer time keratinocytes take to migrate through the ossified portion of the terminal phalanx in distally amputated digits.

Based on these observations, we hypothesized that physiological amputation is a general tissue repair process necessary for re-epithelialization, unrelated to digit tip regeneration.

### Blastema-like tissue is observed in proximally and distally amputated digit tips

**Figure 3:**
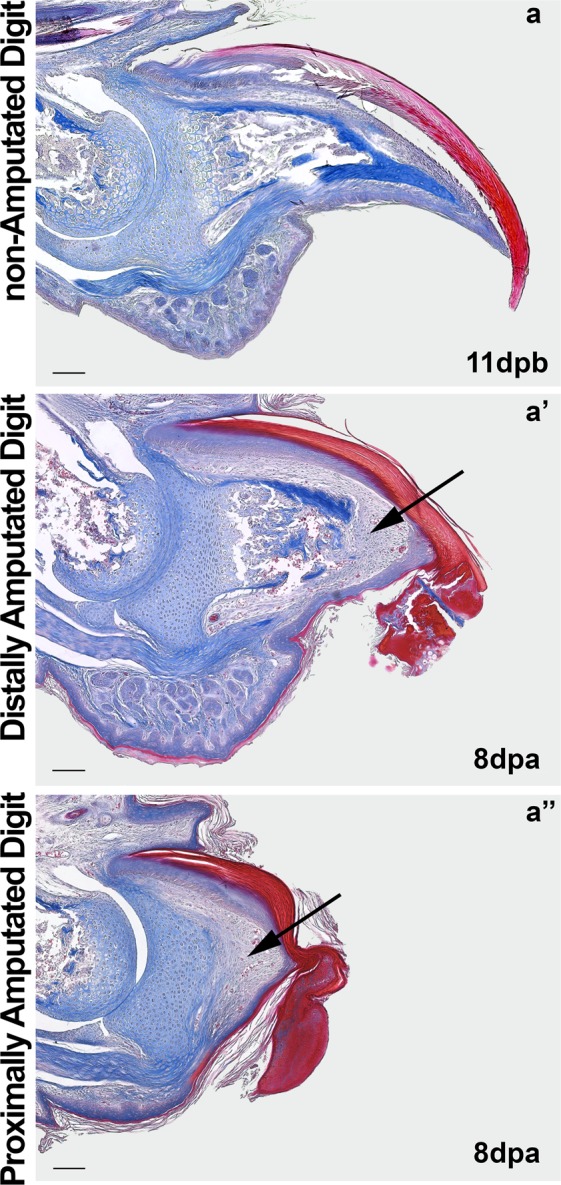
Histological characterization of the mesenchymal and bone tissue after distal and proximal amputation. **a**, non-amputated digit at 11 dab used as control for comparison of the digit tip morphology. An area of vascularized loose connective tissue can be seen at the distal end of the digit between the amputated bone, the nail bed and the regenerated epidermis at distally (a’, arrow) and proximally amputated digits (a”, arrow). The ossified portion of the distal phalanx with the underlying periosteum is partially preserved in the distally amputated digit (a’, arrowhead), while both are completely removed in the proximally amputated digit (a”). Mallory trichrome stain (Scale bars, 100 μm). Amputations performed at 3 days after birth (dab).

The existence of a real blastema during mouse digit tip repair has always been questioned^19,28^. Some authors suggest that in adult primates and rodents, digit tip regeneration occurs in the absence of blastema^7,9,19,20^, or through a process called non-blastemal epimorphic regeneration^18,21,22^. Our histological analyses showed a mesenchymal tissue triangularized between the amputated bone, the nail bed and the regenerated dermis (arrow at Fig. 3a’ – a”). Note that this mesenchymal tissue can be observed in both types of amputations (Fig. 3a’ – a”). This highly vascularized tissue is gradually replaced by bone tissue in distally amputated digits with further development.

### Distal intramembranous ossification in distally amputated digits, not in proximally amputated digits, reconstitute digit tip

**Figure 4:**
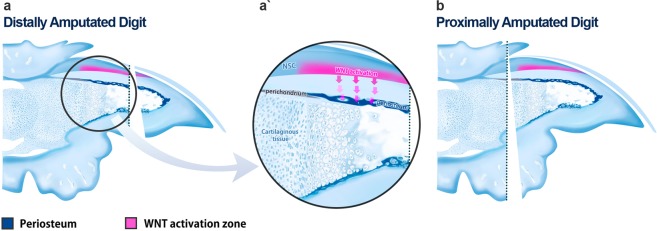
Proposed model for differential tissue repair after digit tip amputation. Distal amputations preserve the Wnt activation zone (pink) and bone progenitor periosteal cells (darkest blue), which are important for distal appositional bone growth during development and after distal amputation (**a**, a’). Proximal amputation removes the Wnt activation zone and the periosteum of the digit tip (**b**). Without Wnt activation and periosteum, no distal appositional bone regrowth is expected to occur. If present, these structures will promote distal appositional bone regrowth, nail bed extension; leading to the close to normal morphology observed in distally amputated digits.

The terminal phalangeal bone is the most prominent structure of the mouse digit tip, its claw form shapes the final format of the digit tip. After distal amputation, the growth of this bony structure is crucial for the aesthetic and functional re-establishment of the amputated digit. Thus, we performed careful observations of the bone growth process in distally and proximally amputated digits in histological preparations, which evidence bone and cartilage tissues and find intriguing similarities and differences. First and most importantly, we observed that proximal and distal amputations differ in the bone context mainly by the amount of ossified portion of the terminal phalanx. While proximal amputation eliminates the ossified portion entirely (Fig. 3a”), distal amputation eliminates it only partially (arrowhead, Figs 3a’ and 4a). This difference could have a major impact on the outcome of both amputations. Bone pieces are surrounded by the periosteum, a mesenchymal tissue that is the main source of bone progenitor cells in mammals^34,35^. On the other hand, bone progenitor cells are the main drivers of regeneration of the phalangeal bone^15,36^. The periosteum is the main determinant of seasonal antler regeneration in deer^37^.

Recent evidence suggests a determinant role of the nail organ on bone growth during digit tip regeneration^17,27^. Interestingly, the signal derived from the nail affecting mouse digit tip regeneration was identified as the Wnt signaling pathway^17^, which is the main activator of periosteum-based appositional ossification^38–40^. Furthermore, some authors^13,14^ demonstrated that bone regrowth in distally amputated digits occur solely due to appositional ossification at the distal region of the amputated digit. Moreover, when implanted in mouse digits amputated at the medial phalanx, the nail organ is capable of inducing bone growth^41^.

Taking together, these observations led us to suggest the following scenario for tissue repair after digit tip amputation (Fig. 4): when mice digits are amputated at distal levels (elimination of up to 30% of the digit tip), the nail epithelium (as the source of osteogenic signals) and the periosteum (as the source of osteoprogenitor cells) are preserved in significant amounts (Fig. 4a – a’). Wnt-expressing nail epithelium (expressing the Wntless gene), necessary and sufficient to promote distal growth of the terminal phalanx after digit amputations^17^, act as a source of bone-inducting signal on mesenchymal cells surrounding the terminal phalanx. The distal and dorsal most position of this osteogenic signal will promote distal appositional bone growth in the amputated phalanx. As the terminal phalanx extends distally the nail epithelium will also extend distally, culminating in the formation of the claw-shaped mouse digit tip. When amputations are made on proximal levels (elimination of over 50% of the digit tip), the region of Wnt-secreting nail epithelium^17^ and the periosteum containing Wnt-responsive mesenchymal cells are both eliminated (Fig. 4c). The lack of distal appositional ossification will then grant the characteristic of regeneration failure in proximally amputated digits. Therefore, the regeneration of the amputated digit would be a result of the maintenance of the process of distal appositional growth, instead of a regenerative response induced upon amputation.

Corroborating the hypothesis of maintenance of the homeostatic processes after distal amputations, there are reports of regenerated digit tips that never re-establish normal length after amputation^13^. This phenomenon can be explained by our model. First, distal amputation eliminates the distal-most ossified portion of the phalanx (Supl. Fig. 1, line b), originally formed by endochondral ossification during digit tip development. Second, digit tip regrowth occurs only by distal appositional ossification. Therefore, we propose that the portion of bone length missing in the regrown distally amputated digits is the portion formed by endochondral ossification and that the final phalangeal length is determined by the continuity of the distal appositional ossification, once tissue homeostasis is established after amputation.

Another important evidence to be considered in our model is the treatment of proximally amputated digits with beads containing bone morphogenetic protein (BMPs) molecules promoting phalangeal regrowth in mice^25,42^. This observation could suggest the induction of an innate regenerative capacity in mice digits. However, the authors report that the longitudinal regrowth observed in these digits occurs by endochondral ossification, implying that BMP7-induced regeneration recapitulates digit tip development. BMP molecules are well known for their osteogenic capacity^43,44^, being capable of inducing bone growth when administered in many different cellular contexts^45,46^. It is possible that the digit re-growth after amputation achieved by BMP treatment resembles a regenerative response exactly because it targets the main structure important to this outcome, the phalangeal bone. Therefore, we believe that it is possible to obtain close to normal external morphology by inducing longitudinal bone growth in the terminal phalanx of amputated digits, either by a nail-dependent distal appositional ossification or by exogenous BMP-dependent endochondral ossification.

### Implication in human health

The control over the process of limb regeneration has been one of the most tempting achievements in human history. Limb losses by war, diseases or accidental damages affect 185.000 people annually in the United States^47^. Despite the existence of many reports documenting digit tip regeneration, most clinical studies in humans are based on the external reconstitution, exploring only cosmetic and functional observations of the digit^7,48,49^. Furthermore, these studies^20,50^ lack the reporting of periodic radiographs of the digit showing the morphology of the phalanx and the external aspect of the digit at the time of amputation until complete digit recovery^14^.

The main implication of our findings, together with the literature discussed in this study, is on defining mouse digit tip as a model system for epimorphic regeneration. Our conclusions favour a view that digit tip regrowth after distal amputation is controlled mainly by a tissue specific intrinsic capacity of repair after a lesion, together with the maintenance of the endogenous distal appositional ossification process. If this hypothesis is correct, any source of osteogenic signal that promotes longitudinal bone regrowth after amputation would reproduce the aesthetical and functional characteristics of the digit tip. In line with this hypotesis, a recent study stimulated bone and joint formation in mouse digits by providing BMP2 to induce bone growth and BMP9 to promote cavitation and chondrogenesis^51^. Therefore, we can speculate that distal bone regrowth could be expected in humans, children or adult, after distal amputation and foresee that mechanisms to regenerate joint structures will be the main goal of regenerative medicine for limb and digit regrowth.

## Material and Methods

### Amputations and animal handling

We used postnatal day 3 neonates C57BL/6J for the amputations. They were anesthetized in ice and the central digit of the right and left hind limbs were amputated. The amputations were performed using microdissection scissors along the proximal third of the nail bed, as previously described by one study^13^. The distal amputation level was the phalangeal bone tissue, while the proximal amputation level was the phalangeal cartilage tissue. This study was performed with the approval and in accordance with the Ethics Committee on the Use of Animals of the Universidade Estadual de Campinas, protocol no. 2659-1.

### Histology and Histological Stain

The digits were collected in times of interest and fixed with 4% paraformaldehyde for 16 hours at 4 °C, dekeratinized in 1% KOH according to one study^13^, decalcified in a solution containing 10% formaldehyde, 8% formic acid and 1% methanol and finally embedded in paraffin. Seven μm thick sagittal slices were cut and stained with Mallory triple stain^52^.

## Supplementary information

Supplementary Figure 1
