# Supplementary material for: Bone growth as the main determinant of mouse digit tip regeneration after amputation
Source: Sci Rep. 2019 Jul 4;9:9720. doi: 10.1038/s41598-019-45521-4 (PMC6609708; doi:10.1038/s41598-019-45521-4)
Supplement: Supplementary file 1 — Supplementary Figure 1 [file 41598_2019_45521_MOESM1_ESM.pdf]

Bone growth as the main determinant of mouse digit tip regeneration after amputation

Sensiate LA, Marques-Souza H

Supplementary Figure 1

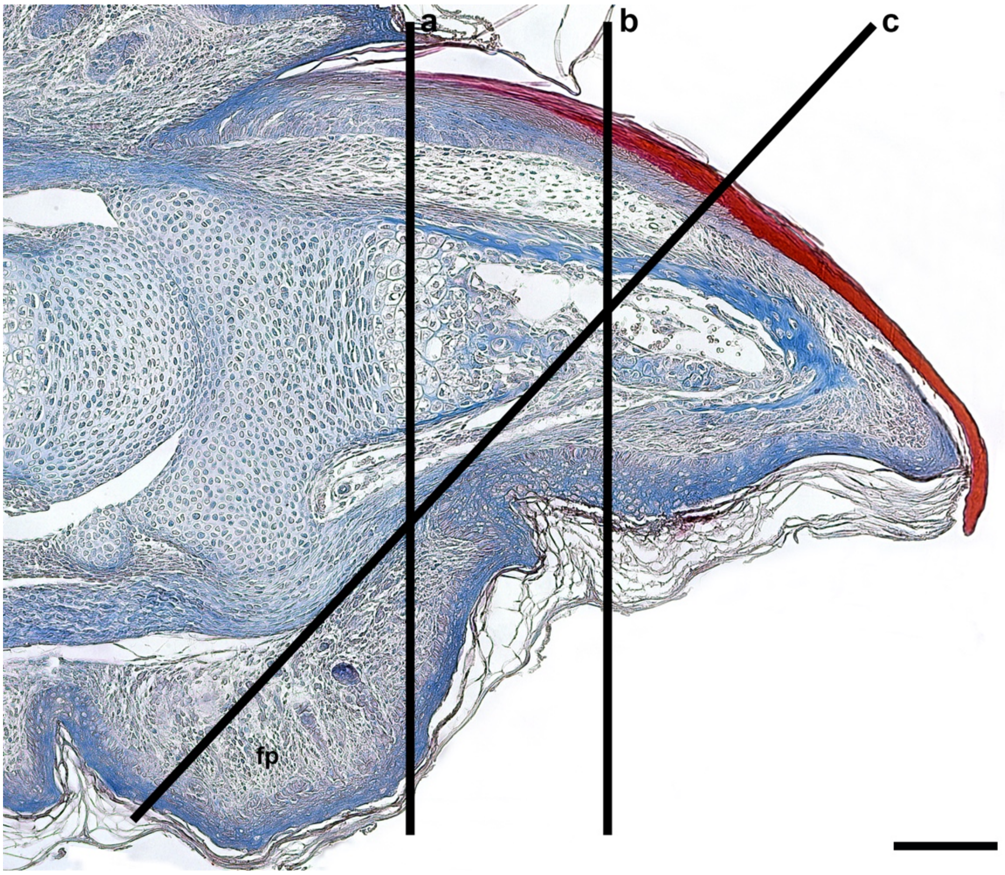

Supplementary Figure 1: Schematic view of the amputation planes used in this work shown in a sagittal histological section of a three-day old mouse digit tip. Proximal amputation that removes ~ 70% of the distal phalanx (a). Distal amputation that removes ~ 30% of the distal phalanx (b). Oblique distal amputation that removes ~ 30% of the distal phalanx and most of the fat pad (c). Mallory Trichrome stain. fp: fat pad. (Scale bar, 100µm.)
